# The Multiple Roles of Lactate in the Skeletal Muscle

**DOI:** 10.3390/cells13141177

**Published:** 2024-07-10

**Authors:** Bianca Bartoloni, Michele Mannelli, Tania Gamberi, Tania Fiaschi

**Affiliations:** Dipartimento di Scienze Biomediche, Sperimentali e Cliniche “M. Serio”, Università degli Studi di Firenze, 50134 Firenze, Italy; bianca.bartoloni@unifi.it (B.B.); michele.mannelli@unifi.it (M.M.); tania.gamberi@unifi.it (T.G.)

**Keywords:** lactate, GPR81 receptor, muscle renewal

## Abstract

Believed for a long time to be merely a waste product of cell metabolism, lactate is now considered a molecule with several roles, having metabolic and signalling functions together with a new, recently discovered role as an epigenetic modulator. Lactate produced by the skeletal muscle during physical exercise is conducted to the liver, which uses the metabolite as a gluconeogenic precursor, thus generating the well-known “Cori cycle”. Moreover, the presence of lactate in the mitochondria associated with the lactate oxidation complex has become increasingly clear over the years. The signalling role of lactate occurs through binding with the GPR81 receptor, which triggers the typical signalling cascade of the G-protein-coupled receptors. Recently, it has been demonstrated that lactate regulates chromatin state and gene transcription by binding to histones. This review aims to describe the different roles of lactate in skeletal muscle, in both healthy and pathological conditions, and to highlight how lactate can influence muscle regeneration by acting directly on satellite cells.

## 1. Lactate as a Metabolite and Signalling Molecule

**Figure 1:**
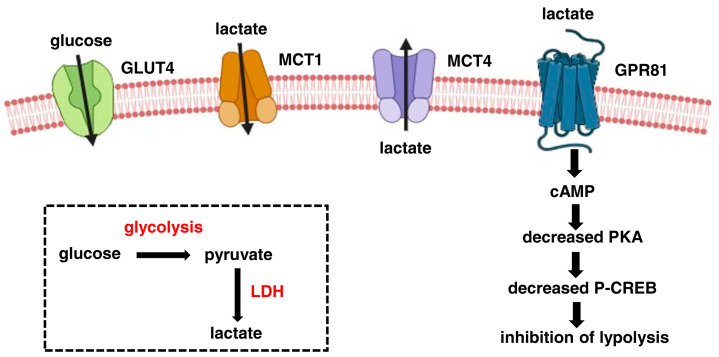
The route of lactate in the skeletal muscle. Glucose (which enters in the muscle through the GLUT4 transporter) is oxidised in two molecules of pyruvate during glycolysis. In anaerobic and in energy-demand conditions, lactate dehydrogenase (LDH) transforms pyruvate into lactate (as shown in the rectangle). Lactate can shuttle between the cytoplasm and the extracellular environment through the two transporters, MCT4 (out of the cell) and MCT1 (within the cell). Extracellular lactate can bind to the specific receptor GPR81. Ligand binding leads to the activation of inhibitory G proteins and the decreased production of cyclic AMP (cAMP). In turn, Protein Kinase A (PKA) activity and phosphorylation of CREB transcription factor are reduced, thus leading to decreased lipolysis. The figure was created with biorender.com.

Skeletal muscle is a dynamic tissue that plays a crucial role in the body’s locomotion, posture, and energy metabolism [1]. The tissue is primarily responsible for energy expenditure, metabolic adaptations, homeostasis, glucose metabolism, and lactate production [2]. The production of lactate in skeletal muscle is the result of lactic fermentation, which is activated during intense anaerobic exercise [3]. During this process, glucose is metabolised through glycolysis to produce pyruvate, which in hypoxic conditions or situations of high energy demand, is converted to lactic acid by lactate dehydrogenase (LDH). This reaction provides a fast alternative pathway for the regeneration of oxidised NAD to sustain glycolytic and ATP production [4]. LDH is a tetrameric enzyme formed by the association of two different subunits, M (muscle) and H (heart), differently expressed and associated in diverse tissues. Skeletal muscle expresses the M subunit that catalyses the conversion of pyruvate in lactic acid, while the heart contains the H subunit that is dependent on aerobic metabolism [5]. Lactate is extruded in a 1:1 ratio with hydrogen ions by facilitated diffusion through the monocarboxylate transporter 4 (MCT4). Once in the interstitial space or bloodstream, lactate can be co-transported with a hydrogen ion by monocarboxylate transporter 1 (MCT1) into the oxidative fibres, where it can be converted to pyruvate [6] (Figure 1). In rat L6 myotubes, MCT1 expression appears to be controlled by lactate that induces *mct1* gene transcription through a mechanism involving reactive oxygen species formation [7]. 

The metabolic role of lactate can be summarised as two crucial functions: (i) it is a source of energy; (ii) it is the main gluconeogenic precursor. Lactate exchanges within and between cells are referred to as “intracellular” lactate shuttles, including cytosol–mitochondrial and cytosol–peroxisome exchanges and “cell-to-cell” lactate shuttles [8]. The role of lactate transport in metabolism has been extensively investigated. Although lactate production as an end point of aerobic glycolysis represents a critical point known as the Warburg Effect in cancer [9], scientific evidence shows that aerobic glycolysis leading to lactate production also occurs under high-stress conditions, such as high-altitude exposure, trauma, sepsis, pancreatitis, and heart failure [10]. Lactate is now rightly and strongly considered to be a valuable metabolite, replacing the traditional view that interpreted increased lactate levels as evidence of an anaerobic condition at the cellular level [8]. 

**Figure 2:**
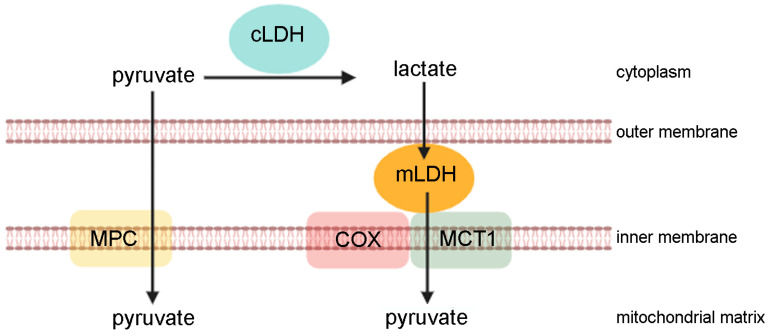
The lactate in the mitochondria. Lactate produced in the cytoplasm by cytoplasmic LDH (cLDH) enters the intermembrane space, freely crossing the outer mitochondrial membrane. Here, the mitochondrial LDH (mLDH) transforms lactate in pyruvate, which enters the mitochondrial matrix through the MCT1 transporter. MCT1, localised in the inner mitochondrial membrane, together with cytochrome oxidase (COX) and mLDH constitute the so-called “lactate mitochondrial oxidation complex”. Cytoplasmic pyruvate directly enters the mitochondrial matrix, passing through the mitochondrial pyruvate complex (MCP) located in the inner mitochondrial membrane. In the mitochondrial matrix, pyruvate becomes the substrate of the pyruvate dehydrogenase complex that catalyses the conversion of the pyruvate into acetyl CoA. The figure was created with biorender.com.

In skeletal muscle, lactate can be directly oxidised in mitochondria. In 1986, Brooks suggested the “lactate shuttle theory”, according to which lactate is up taken by cellular organelles, such as mitochondria or peroxisome, or secreted into the bloodstream and used by organs as a fuel [4,10]. The oxidation of lactate in mitochondria has been demonstrated by several approaches performed on cells and tissues (such as magnetic resonance spectrometry, confocal microscopy, and immunohistochemistry) [11,12]. Extensive studies in healthy human subjects show that the main lactate oxidation site is the mitochondrial lactate complex. Here, lactate is converted by mitochondrial LDH to pyruvate that is driven towards the Krebs cycle. This process is very useful in skeletal muscle since the ability to use lactate as an energy source provides an effective mechanism for ATP regeneration in the presence of high energy demand, such as during high-intensity exercise. The mitochondrial lactate oxidation complex is composed of mitochondrial LDH that is anchored to the outer side of the inner mitochondrial membrane and associated with cytochrome oxidase (COX) and mitochondrial MCT1. The exergonic redox change in cytochrome oxidase during mitochondrial electron transport is coupled with lactate oxidation. Finally, MCT1 transports pyruvate across the inner mitochondrial membrane [12] (Figure 2). 

During muscle contraction, the metabolic rate of skeletal muscle rises exponentially, and the huge consumption of ATP is buffered by the modulation of glycolysis and glycogenolysis rates [13]. In the muscles and arterial blood of resting healthy humans, lactate concentration approximates 1.0 mM [13]. However, lactate concentration is subjected to considerable fluctuations with a significant change in the lactate/pyruvate ratio that dynamically affect the NAD+/NADH ratio. Therefore, lactate production has a significant impact on metabolic regulation and cellular redox. In this regard, lactate can affect the cellular production of reactive oxygen species (ROS) through both enzymatic and non-enzymatic pathways, primarily by affecting ROS production through mitochondrial respiration [14]. In this regard, mitochondrial lactate metabolism is involved in the generation of ROS, mainly hydrogen peroxide, via a flavin-dependent lactate oxidase (LOX) pathway.

Most of the lactate (75–80%) is disposed within the tissue or released and taken up by the working muscle [15], heart [16], brain [17], and liver for gluconeogenesis [18]. In addition to oxidative fuel, lactate is important in supplying carbon to gluconeogenesis. In this regard, the skeletal muscle and liver are linked by the so-called “Cori cycle”, an organ–organ lactate shuttle. The cycle ensures that the liver is supplied with lactate that is directed into gluconeogenesis after being converted to pyruvate by hepatic LDH. 

Molecules produced during physiological cellular metabolism were for a long time considered exclusively to be a metabolic waste [19]. In recent years, their role has been reassessed, since these molecules can orchestrate a variety of biological processes through their binding to receptors or specific transporters. Lactate is among these multifunctional metabolites, acting as an intracellular signalling mediator and as an extracellular ligand [20,21]. Lactate displays a signalling function through binding to the lactate-activated G-protein-coupled receptor GPR81, also termed Hydroxycarboxylic Acid Receptor 1 (HCA1) [22]. This receptor is located in the plasma membrane, and its presence has been observed in other intracellular organelles, such as mitochondria, thus implying a role for GPR81 in the trafficking of lactate between the plasma membrane and intracellular compartments [23]. Predominantly expressed in the adipose tissue, GPR81 is present in the skeletal muscle, liver, spleen, kidney, and brain [24,25]. A lactate concentration between 0.2 and 1.0 mM results in the activation of the receptor, followed by cyclic AMP (cAMP) downregulation and the inhibition of protein kinase A (PKA)-mediated signalling [22]. Recent studies showed that lactate-induced GPR81 activation signals through a non-canonical, cAMP/PKA-independent pathway involving beta-arrestin, a GPR81 adaptor protein. This leads to the inhibition of TLR-4 and NOD-like receptor pyrin-domain-containing protein 3 (NLRP3) inflammasome, which mediates the production of proinflammatory mediators such as IL-1 beta and IL-18 [26,27]. Increasing evidence has shown that lactate plays different roles in various pathophysiological conditions, including inflammation and cancer. The GPR81 receptor mediates the non-metabolic effects of lactate, including the regulation of immune and inflammatory response, active participation in wound healing with increased angiogenesis, decreased adipocyte lipolysis, and neuroprotection [24]. These effects depend on several factors such as lactate concentration, extracellular acidity, and the amount of other nutrients in the cellular microenvironment [28]. Recent reports suggest that lactate produced by aerobic glycolysis has an immunosuppressive effect in the local environment in various disease conditions, including sepsis, cancer, chronic inflammation, and autoimmune diseases [29,30]. Lactate inhibits proinflammatory responses in macrophages in both cAMP-independent and GPR81-independent manner [31]. Lactate increases the production of anti-inflammatory interleukin-10 (IL-10) in dendritic cells and diminishes the production of proinflammatory IL-12 in response to Toll-like receptor (TLR) stimulators [32]. Moreover, lactate can also downregulate TNF alpha, NF-KB, and PTX-3, and upregulates IL-23 in monocytes, and transiently downregulates chemokines [33]. Lactate has also been shown to suppress proliferation and cytokine production in human cytotoxic T cells, thereby decreasing the cytotoxic effect [34,35]. Moreover, lactate is involved in reparative angiogenesis through the recruitment of endothelial progenitor cells, the stimulation of the endothelial cell migration, the activation of procollagen factors, and the enhancement of collagen deposition in the extracellular matrix [36,37]. It has been reported that the oral administration of the GPR81 agonist 3-Chloro-5-hydroxybenzoic acid (CHBA) to mice significantly increases the weight of plantaris and soleus muscles, and this effect is associated with the enhanced phosphorylation of ERK1-2 proteins [38]. In human skeletal muscle, GPR81 is significantly expressed mainly in type II glycolytic fibres, compared with type I oxidative fibres. Type II fibres produce the highest amounts of lactate, suggesting that lactate may function as an autocrine signalling molecule [39]. Upon ligand binding, GPR81 inhibits cAMP-dependent protein kinase (PKA) signalling and lipolysis (Figure 1). The lactate-induced inhibition of lipolysis, firstly shown in adipose tissue, has also been reported in mouse skeletal muscle [40]. One well-defined target of PKA is the cAMP-response element binding (CREB) protein. Consequently, the binding of lactate to GPR81 results in lower PKA activity, which leads to reduced CREB phosphorylation. Although exercise is known to increase lactate production, the effects on CREB phosphorylation are unclear so far [39]. In addition to regulating lipolysis, lactate has also been shown to stimulate the expression of genes involved in lactate and energy metabolism in skeletal muscle, as evidenced by Hashimoto et al. [7]. However, it is not yet known whether the effect of lactate in the regulation of gene expression is GPR81-dependent.

## 2. Lactate in Muscle Renewal 

Skeletal muscle is characterised by a high regenerative capacity due to the presence of satellite cells (SC). Located between muscle basal lamina and sarcolemma, SC plays a key role in the regenerative process of adult skeletal muscle and in the maintenance of muscle mass. SC are normally in a quiescent state characterised by a relatively low energy demand. Following skeletal muscle damage, SC are rapidly activated to proliferate and differentiate to regenerate muscle tissue [41]. The metabolic profile undergoes significative modifications in quiescent, activated, and differentiated SC, suggesting that metabolic changes may influence the SC’s fate [42]. Recent data suggest that external factors in the muscle microenvironment, such as metabolites, could play a key role in SC activation. In this regard, lactate has been proposed as an excellent candidate. A lactate-dependent mechanism that indirectly controls SC’s fate has recently been reported. During ischemia, endothelial cells increase the secretion of lactate, exploiting their glycolytic activity. Extracellular lactate targets macrophages, inducing their polarisation towards an M2-like phenotype. These lactate-polarised macrophages enhance the expression of vascular endothelial growth factor (VEGF) and stimulate the proliferation and differentiation of muscle progenitor cells. These events support muscle revascularisation and regeneration after SC activation [43]. 

In vitro, lactate acts as a pro-myogenic factor, regulating the proliferative and differentiation rate of murine C2C12 myoblasts. The differentiation of myoblasts is induced by 10 mM lactate by increasing the expression of the myogenic determination protein (MyoD). In mice, the peritoneal injection of lactate provokes enhanced regeneration and fibre hypertrophy in regenerating muscles, showing that lactate also acts as myogenic factor in vivo [44]. Moreover, skeletal muscle regeneration is associated with the upregulation of the lactate transporter MCT1, which is useful for lactate uptake by muscle cells [45]. Extracellular lactate promotes C2C12 myotube hypertrophy by activating anabolic intracellular signals in a ROS-dependent pathway [46]. In mice, muscle regeneration in the cardiotoxin-treated tibialis anterior is promoted by the oral intake of lactate that induces the increase in Pax7-positive cells. The administration of 20 mM lactate during myogenesis induces the formation of myotubes characterised by increased diameter, length, and protein content in comparison with the control counterpart [38,47]. The positive effects of lactate on muscle differentiation occur in the early stage of differentiation, while the treatment of myoblasts with 20 mM lactate seems to delay the late phase of myogenesis [46,48]. This effect is due to the lactate-dependent downregulation of p38 MAPK phosphorylation, associated with decreased trimethylation of lysine 4 of histone 3 and the reduced expression of Myf5, myogenin, and myosin heavy chain (MHC) proteins. The same patterns have been observed in human skeletal muscle biopsies after training [48]. 

## 3. Protein Lactylation in Skeletal Muscle

In 2019, a novel type of protein post-translational modification was reported for the first time. The authors showed that the lysine residue of histones can bind to lactate, thus generating so-called “protein lactylation”. Histone lactylation acts as an epigenetic modulator that directly stimulates gene transcription, and a total of 28 lysine lactylation sites have been identified on the core histones in both human and mouse cells [49]. New lines of evidence have uncovered that lactate induces histone lactylation in the promoters of the profibrotic genes in macrophages, leading to the upregulation of epigenetic modifications in the fibrotic lungs [50]. Moreover, lysine lactylation in the brain is regulated by neural excitation and social stress [51]. In skeletal muscle, a correlation between histone lactylation and myogenesis has been reported both in vitro and in vivo. Lactate promotes the lactylation of lysine 9 in H3 histone, leading to increased Neu2 transcription and myogenesis [52]. Moreover, protein lactylation is involved in the maintenance of cell homeostasis in skeletal muscle during intense exercise. This process involves the lactylation of Vps34, a catalytic subunit of the Phosphatidylinositol 3 kinase (PI3K) complex, which phosphorylates phosphatidylinositol (PI) to produce phosphatidylinositol 3-phosphate [PtdIns(3)P]. Lactylated Vps34 increases lipid kinase activity to enhance autophagy and endolysosomal degradation, thus promoting muscle homeostasis [53]. Protein lactylation in skeletal muscle correlates with obesity and higher levels of plasma lactate. This effect appears more pronounced in obese females in comparison with lean females and male individuals [54]. Moreover, protein lactylation is associated with an increased phosphorylation on serine 636 in IRS-1, an effect already shown in insulin resistant conditions [55,56,57], as in the human skeletal muscle cells derived from type 2 diabetic donors [55]. In contrast, insulin sensitivity is negatively associated with skeletal muscle lactylation [54].

## 4. Lactate and Physical Exercise

In past years, skeletal muscle was primarily considered the site of lactate production during contraction, associated with poor muscle oxygenation and fatigue. However, skeletal muscle plays an important role not only in the production of lactate but also in its clearance [58]. Moreover, lactate generated during exercise is involved in the energy metabolism rate, mediating exercise adaptations and interorgan communication [59], and it can be considered an important source of carbohydrates in competition with glucose [60]. 

Skeletal muscle produces large amounts of lactate, mainly for two reasons: (i) at the start of moderate-intensity exercise, the rate of glycolysis is massive and faster in comparison to the oxidative phosphorylation pathway; (ii) the maximum glycolytic capacity exceeds the maximum oxidative ability [61]. Lactate production and consumption change during exercise, as shown in a typical blood lactate curve. Lactate is produced and used as fuel during skeletal muscle contraction, and most of the formed lactate is absorbed and oxidised by the producing fibre [61]. During the initial transition phase, between rest and exercise, a sudden rise in energy response occurs and blood lactate levels moderately increase. A rise in ATP demand by working muscles activates glycolysis, leading to increased pyruvate production. In this phase, most of the produced pyruvate is converted to lactate, thus enhancing circulating lactate levels, since no adequate increase in heart rate and capillary dilatation for the delivery of oxygen to contracting muscles occurs. When the cardiovascular response to exercise happens, more oxygen is delivered to skeletal muscle and blood lactate levels decrease and stabilise. In a subsequent phase of training, when exercise intensity increases, the pyruvate production exceeds the capacity of glycolytic fibres to oxidise pyruvate. As a consequence, pyruvate is reduced to lactate by LDH-A. An increase in circulating lactate during training is dependent on oxygen delivery, mitochondrial capacity, and the ability of other cells to take up and use lactate [10,62]. 

Intense physical exercise is associated with so-called “lactic acidosis”. Although the production of lactate is associated with hydrogen ion (H^+^) formation, the exact origin of this acidosis is still debated. It has been argued that non-mitochondrial ATP turnover could be the source of H^+^ [63], while others have hypothesised that the maintenance of electroneutrality occurs between lactate (negatively charged) and the H^+^ provided by water dissociation [64]. Acidosis seems to jeopardise muscle contraction, leading to fatigue, a rapid deterioration of contractile function, and eventually to cessation of exercise. The accumulation of lactic acid in muscles has been always recognised as the main cause of muscle fatigue. In humans, the concentration of intracellular lactate increases during intense exercise, up to 30 mM, in association with a small decrease in intracellular pH (about 0.5 units) [65]. However, acidification does not affect the reduction rate in fibre shortening due to muscle fatigue, since lactic acid plays a metabolic buffering role as well as oxidised NAD regeneration, rather than the onset of acidosis [59,66]. 

In sport medicine, the maximum level of physical effort not associated with lactate and hydrogen ion accumulation in the blood and muscles is called the “anaerobic threshold” or “lactate threshold”. This threshold refers to the intensity and duration of physical activity without changing from aerobic to anaerobic condition. The lactate threshold is considered an excellent predictor of performances, and it is enhanced by intense exercise, which in turn increases performance. As the muscle respiration rate decreases, lactate produced during muscle contraction is released into the blood, even after exercise. Subsequently, lactate is transported by blood circulation to the liver, where it becomes a preferable substrate for hepatic glucose synthesis via a process called the Cori cycle [67,68]. Most of the lactate produced from active muscle is released and taken up by exercising and non-exercising muscles, as well as the heart and brain, where it is oxidised and used as an energy substrate for the neurons [69,70]. Indeed, lactate produced during exercise is considered to influence brain cell function. Hence, the lactate accumulated during exercise is not merely a waste product due to oxygen deficit, since it acts in many processes. Lactate is exchanged between various tissues, such as between different muscles and between various fibre types within the contracting muscles, and it is transported to other organs. Among these, in the heart and brain, lactate is used for mitochondrial respiration [71], while in the kidney and liver it is directed to gluconeogenesis [65]. 

## 5. Lactate in the Skeletal Muscle Diseases

Lactate has been associated with various diseases, such as obesity and insulin resistance [72]. Regarding insulin resistance, it is considered one of the main challenges leading to the development of type 2 diabetes [73]. The involvement of lactate in the onset of insulin resistance has been reported in the skeletal muscles of rats, where elevated amounts of plasma lactate suppressed insulin-stimulated glycolysis. This effect was associated with decreased glucose uptake and the alteration of the insulin signalling cascade [74]. 

The increased production of lactate has been reported in cancer cachexia, a devastating pathology induced by oncologic disease that leads to pronounced weight loss and the worsening of the oncologic condition. Muscles of cachectic mice showed enhanced MCT1 levels and significantly increased lactate/pyruvate ratio, suggesting the high production and release of lactate by the tissue [75]. Myotubes, as well as cachectic mice, showed elevated amounts of lactate in cachectic conditions associated with decreased oxygen consumption [76,77]. The cachectic phenotype in myotubes is accompanied by a metabolic modification towards lactic fermentation. Lactate dehydrogenase inhibition by sodium oxamate impedes the formation of the cachectic condition, thus demonstrating that this metabolic conversion is useful for the onset of the cachectic condition [76]. Moreover, the lactate/pyruvate ratio appears to be decreased in muscles of cachectic mice, due to the increased production of lactate in this condition. The treatment with 2-deoxyglucose, that inhibits glycolysis and impedes the formation of pyruvate, blocks the induction of the cachectic characteristics in mice, thus demonstrating the significant involvement of lactate in the onset of cancer cachexia [78]. Moreover, the ablation of the lactate receptor GPR81 is sufficient to impede the onset of cancer cachexia in both the adipose tissue and skeletal muscle [79], thus highlighting the importance of lactate in the onset and progression of cancer cachexia. 

Genetic mutations in gene-encoding lactate transporter MCT1 or an altered expression of the protein are observed in muscular pathologies. An altered MCT1 level has been reported in McArdle disease [Glycogen Storage Disease Type V; MD), a metabolic myopathy characterised by the absence of glycogenolysis due to a deficiency in muscle glycogen phosphorylase [80]. MD patients show increased MCT1 levels, probably as a compensatory mechanism for energy production in the absence of glycogenolysis [81]. MCT1 is encoded by the solute carrier family 16, with the member 1 [*SLC16A1*) gene organised as five exons interspersed by four introns [82]. Mutations in the *SLC16A1* gene lead to clinical conditions. For example, patients with recurrent and severe ketoacidosis, a pathology that occurs when ketone formation exceeds ketone utilisation, show homozygous and heterozygous mutations in the *SLC16A1* gene [83,84]. Moreover, a missense mutation in the MCT1 gene is responsible for altered lactate transport and muscle injury in humans [85]. Emerging evidence has shown that single-nucleotide polymorphism (SNP) can be used as a promising biomarker of individual genetic background to predict therapeutic responses and prognosis in cancer patients [86]. With regard to this, SNP in the MCT1 gene could be used as a prognostic marker in colorectal cancer patients and as a predicting marker in adjuvant chemotherapy [87].

## 6. Concluding Remarks

The point of view regarding the role of lactate in skeletal muscle has changed significantly over the years. As previously described, lactate has numerous and varied functions in skeletal muscle, in both pathological and healthy conditions. However, many aspects still need to be clarified. Firstly, the role of protein lactylation (not only regarding histones, but proteins in general) must be addressed in depth. An issue to be answered is whether lactate can influence the state of chromatin in all tissues, and whether this epigenetic effect is modified in pathological conditions. Moreover, the question of how and whether lactate binding to non-histone proteins can influence their function remains unresolved. Mass spectrometry combined with the use of primary antibodies that recognise lactate binding to proteins may be useful in this research. Moreover, the role played by the metabolite in skeletal muscle will need to be analysed widely. Increasing evidence highlights that the lactate receptor GPR81 could be involved in the onset and/or maintenance of muscle diseases (for example, in the onset of muscle wasting induced by a neoplastic pathology). No effective GPR81 antagonists, aiming to avoid the negative effects of lactate as a signalling molecule, are available so far. From this point of view, the discovery of new receptor inhibitors could contribute to the amelioration of those pathologies in which lactate plays an important role as signalling molecule.

## Data Availability

No new data were created or analysed in this study. Data sharing is not applicable to this article.
